# Photodegradation of Acidolysis Lignin from BCMP

**DOI:** 10.3390/molecules13123129

**Published:** 2008-12-15

**Authors:** Mohammad Azadfallah, Seyed Ahmad Mirshokraei, Ahmad Jahan Latibari

**Affiliations:** 1 Wood and Paper Science and Technology Department, University of Tehran, Karaj, Iran; 2 Department of Chemistry, Payam-e-Nour University, Tehran, Iran; E-mail: mirshokr@pnu.ac.ir (S-A. M.); 3 Wood and Paper Science and Technology Department, Islamic Azad University, Karaj Branch, Karaj, Iran; E-mail: latibari_24@yahoo.com (A-J. L.)

**Keywords:** Lignin, Photodegradation, BCMP, ^1^H-NMR

## Abstract

A mild acidic dioxane extraction method was employed to isolate lignin from hardwood bleached chemimechanical pulp (BCMP). The isolated lignin was then purified and undergone elemental analysis. To study the photodegradation behavior, the lignin samples were impregnated onto the Whatman filter papers and irradiated with UV light for various periods. The photolyzed lignin was then recovered and analyzed by ^1^H-NMR spectroscopy. Phenylpropane-based formula (C_9_) of CMP pulp lignin and the photolyzed samples were then established with elemental analysis and ^1^H-NMR spectroscopy data. The results indicated that the benzaldehyde and benzoic acid type compounds were the main photodegradation products of BCMP lignin. The lignin photodegradation probably involved the degradation of phenylcoumaran units. Irradiation also increased the phenolic hydroxyl group content and decreased that of methoxyl groups, due to demethoxylation. The degrees of aromatic ring condensation were increased upon continuing the irradiation time, which imples the formation of condensed structures in photolyzed lignin.

## Introduction

Lignin-rich papers made from mechanical pulps turn yellow when exposed to light and this phenomenon is a major barrier for using mechanical pulps in papers that require long-term brightness stability. So their utilization is usually limited to making newsprint and short-life advertising papers [1, 2].

The photoyellowing process of mechanical pulp is believed to be initiated by chromophoric groups found in lignin, including catechols [3], aromatic ketones, coniferaldehyde, stilbenes, and conjugated phenolics [4]. These functional units absorb near UV light and initiate a series of complex chemical reactions that ultimately yield visible chromophores, which discolor the pulps. Recently dibenzodioxocin structures which include a large part of the biphenyl structures of lignins [5,6,7], were also shown to be photoreactive [8, 9], due to the presence of both photoreactive α-O-4 and β-O-4 linkages.

The photodegradation mechanisms have not completely been elucidated, but three main reaction pathways have been identified, namely, the phenoxy pathway, the phenacyl pathway and the ketyl pathway. Phenoxy radicals, the key intermediates, are formed through these pathways. Colored species like O-benzoquinones, formed by oxidation of phenoxy radicals [10] and the hydroquinone/ benzoquinone redox systems have been considered in the coloration process of mechanical pulps [11, 12].However, the overall contribution to the photoyellowing of mechanical pulps by these functionalities, characterization of photodegraded lignin products, and chromophoric structures of photoaged mechanical pulps, still remain as active fields of investigation.

Mixtures of hardwoods are usually used in Iran to produce high yield pulp with a chemimechanical pulping (CMP) process. Recently high pressure size exclusion chromatography (HPSEC) analyses have been used to determine molecular weight changes and distribution of reaction products formed during irradiation of bleached CMP lignin [13]. In order to monitor exactly the light-induced changes in lignin structure, this study was designated to examine the photodegradation of lignin isolated from hardwood bleached CMP pulp using ^1^H-NMR spectroscopy and elemental analysis. This study will increase our basic knowledge about hardwood bleached CMP pulp photochemical behaviors and would pave the way to develop a practical solution to retard photoyellowing.

## Results and Discussion

### ^1^H-NMR spectroscopy

**Figure 1:**
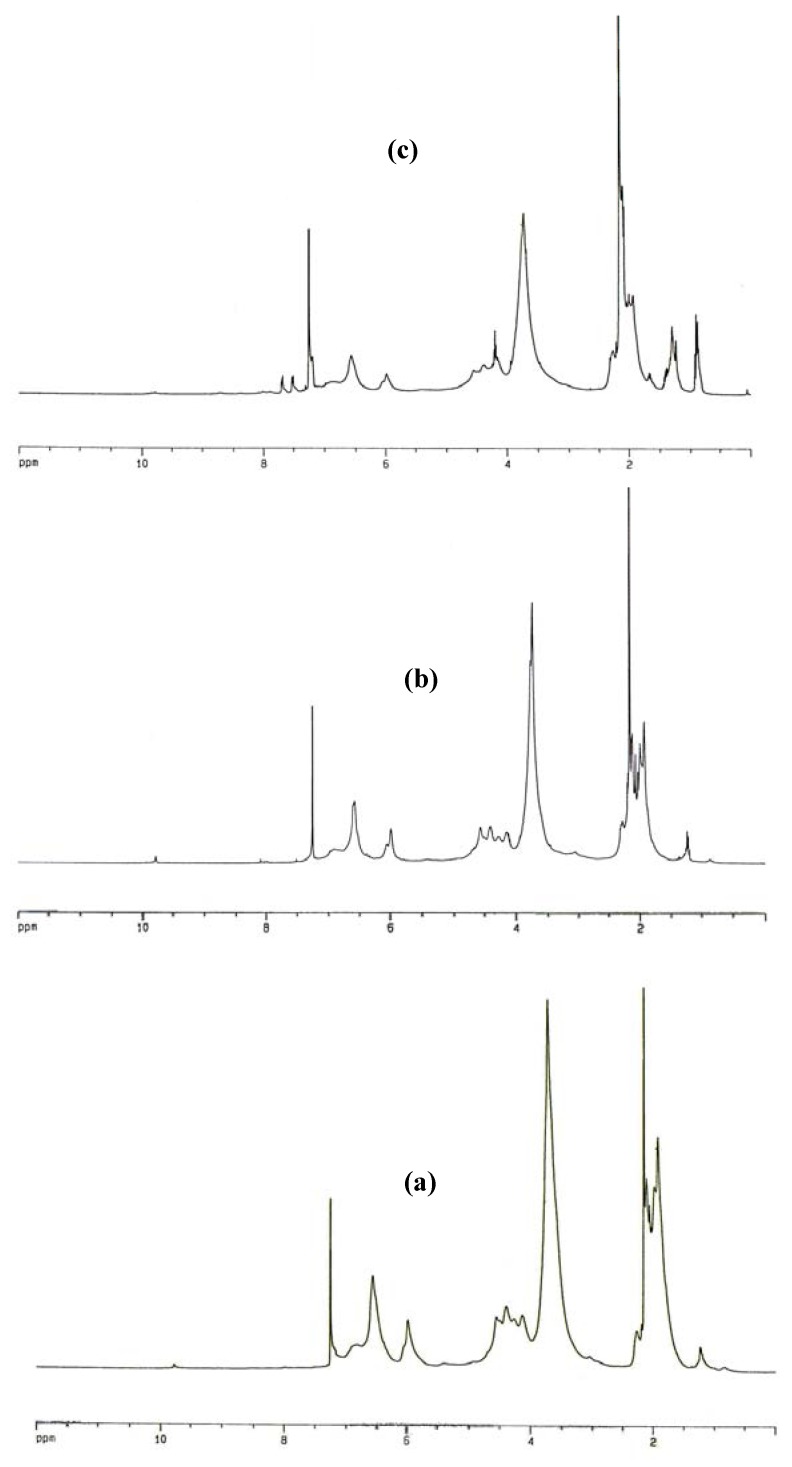
^1^H-NMR spectra of unirradiated BCMP lignin (a) and irradiated lignins for 4h (b) and 120h (c).

The ^1^H-NMR spectra of acidolysis dioxane lignin isolated from BCMP and irradiated lignin samples for 4 h and 120 h are shown in Figure 1. The interpretations of spectra are based on data which reported elsewhere [14,15,16]. The related signals assignments in chemical shift ranges are summarized in Table 1. All spectra normalized to 100. The integration of the signal areas of the spectra is given in Table 2.

Broadening of signals which is obvious from the appearance of the lignin spectra, is thought to be due to a tendency toward rigidity caused by cross linking and large rings in the macromolecular structure of the lignins and to spin-spin splitting effects.

The ^1^H-NMR spectra of the irradiated lignin samples exhibited sharp peaks in the aldehyde proton region (δ = 9.87-9.58 ppm) and on the broad signal in the aromatic proton region (δ = 8.0-6.28 ppm). The peaks were probably due to monomeric aromatic aldehyde and carboxylic acids resulting from the light-induced degradation of the lignin. Vanillin and vanillic acid have been found to be the main low-molecular degradation products formed on irradiation of lignin-rich paper made of softwoods [17]. However, since BCMP pulp has been produced from hardwoods, then oxidation products of hardwood lignin such as syringaldehyde and syringic acid [18] may consist part of the photodegraded products. However no direct measurements were conducted to determine the photodegraded products.

Besides the aldehyde signal of vanillin and syringaldehyde, the formyl proton signal in cinnamaldehyde type units could be observed in the region δ = 9.87-9.58 ppm [14, 15] of the spectra of the irradiated samples. Since the signals all appear to be singlet, then it excludes cinnamaldehyde type groups and are probably due to different benzaldehyde type units and/or monomers.

**Table 1:** The main signals assignment of the ^1^H-NMR spectra of BCMP acetylated lignin [14,15,16].

Chemical shift range ( δ in ppm )	Hydrogen Type
2.2-1.58	Aliphatic acetoxyl region
2.5-2.2	Aromatic acetoxyl region
3.95-3.55	Methoxyl
4.9-4.5	H-α in β-O-4 units
5.8-5.2	H-α in phenylcoumaran (β-5) units
8.0-6.28	Aromatic region
8.5-7.4	H-2 and H-6 in benzaldehyde and benzoic acid
9.87-9.58	Formyl in benzaldehyde units

**Table 2:** The ^1^H-NMR data of unirradiated and irradiated BCMP lignins.

Chemical shift range (ppm)	Irradiation time (hour)				
	0	2	4	24	120
2.2-1.58 ^*^	9.30	9.28	9.20	9.03	8.85
2.5-2.2 ^*^	0.63	0.65	0.7	1.13	1.91
3.95-3.55	31.19	29.73	28.34	24.2	21.5
4.9-4.5	4.69	4.54	4.48	4.16	3.43
5.8-5.2	0.72	0.6	0.58	0.83	0.9
8.0-6.28	10.96	10.35	9.79	7.89	7.36
8.5-7.4	0.05	0.13	0.14	1.43	1.59
9.87-9.58	0.1	0.16	0.14	0.31	0.1
Total ^†^	80.13	80.14	80.2	79.67	78.49

* divided by 3

† consists other neglected δ ranges area.

Long-term 24h and 120h irradiation caused to a relative intensity increase within the aromatic part of δ=8.0-7.4 ppm region of the spectra. In this region mainly H-2 and H-6 in aromatic rings with an α-carbonyl group as well as H-α in cinnamaldehyde-cinnamic acid type units absorb [16]. The largest contribution to this intensity increase then probably comes from the signals of H-2 and H-6 in benzaldehyde and benzoic acid type units, besides other α-carbonyl group containing species.

Intensities of signals at δ = 5.8-5.2 ppm assigned to H-α in β-5/α-O-4 (phenylcoumaran) units, decreased in the spectra of the lignin samples photolyzed up to 4 h irradiation. The decrease is probably a result of α-O-4 cleavage which could be followed by formation of stilbene. This is in agreement with others’ findings [19]. However, with proceeding of irradiation up to 24 h and 120 h, the intensities of signals at this region increased which could be attributed to the high formation rate of condensed structures like phenylcoumaran unit than its degradation at long-term irradiation.

A decrease of the intensities of the signals of H-α in β-O-4 units at δ = 4.9-4.5 ppm was also observed in spectra of irradiated samples. This indicates that α-CHOH groups are transformed, probably by oxidation to carbonyl groups. This is supported by the intensity increase of low-field part of the aromatic signals especially in spectra of long-term irradiated lignin.

### Elemental analysis and developing C_9_-based empirical formulas

The isolated and purified BCMP lignin was also characterized by elemental analysis. These analytical data and the results of the semiquantitative analysis of the ^1^H-NMR spectra of unirradiated and irradiated samples were used to establish empirical formula calculated on phenyl propane (C_9_) basis. These formulas serve to lend emphasis to the fact that the monomeric units comprising lignin have C_9_ carbon skeletons and to underscore the significance of the methoxyl group for both identifying the lignin type and for indicating the contributions of component monomer units [20].

A method developed by Ludwig *et al*. [15] used to determine the number of hydroxyl groups using ^1^H-NMR data of acetate derivatives and elemental analysis data. Aliphatic acetoxyl and aromatic acetoxyl signals permit the estimation of the number of hydroxyl groups. However, one should bear in mind that the aromatic acetoxyl groups in 5-5’ bonded dimeric units appear in the range of aliphatic acetoxyl groups and are not considered in the estimation of the phenolic groups [21].

**Table 3:** Expanded empirical formulas of BCMP acidolysis lignin and irradiated lignin samples based on C_9_.

Irradiation time (hours)	C_9_-based empirical formula
0	C _9_ H_4.867_ O _1.629_ H ar. _1.94_ (OH) ar. _0.112_ (OH) al. _1.64_(OCH_3_)_1.83_
2	C _9_ H_5.207_ O _1.713_ H ar. _1.82_ (OH) ar. _0.113_ (OH) al. _1.61_(OCH_3_)_1.72_
4	C _9_ H_5.477_ O _1.772_ H ar. _1.71_ (OH) ar. _0.121_ (OH) al. _1.59_(OCH_3_)_1.63_
24	C _9_ H_6.127_ O _1.932_ H ar. _1.56_ (OH) ar. _0.188_ (OH) al. _1.51_(OCH_3_)_1.35_
120	C _9_ H_6.344_ O _1.910_ H ar. _1.50_ (OH) ar. _0.318_ (OH) al._1.48_(OCH_3_)_1.20_

From the elemental analysis of isolated bleached CMP lignin (C = 56.95 %; H = 6.2 %; O = 36.4 %) the following atomic ratio was obtained: C_4.17_H_6.15_O_2.28_. The empirical formulas of isolated bleached CMP acidolysis lignin and irradiated lignin samples as shown in Table 3 were then developed in accordance with the procedure described elsewhere [15]. As can be seen from the empirical formulas, the phenolic hydroxyl groups of the photolyzed lignin samples increased, especially after long-term irradiation. The photodegradation of lignin caused the scission of β-O-4 structures resulting in the formation of free guaiacyl and syringyl phenolic units. It seems that some of the quenching of these phenoxy free radicals proceeds via hydrogen abstraction yielding new phenolic units. However, formation of such units is slower than the rate of cleavage of β-O-4 bonds [22]. The formation of new phenolic units is not remarkable given the extreme reactivity of phenoxy radicals in reactions other than simple hydrogen abstraction. Because phenoxy radicals are the products of hemolytic bond scission taking place within the β-O-4 structures, they may further condense or disproportionate to yield chromophoric structures. In addition, the opening of the hydrofuran ring by cleavage of α-O-4 bond in phenylcoumaran units and formation of stilbene structures during irradiation could lead to the formation of new phenolic hydroxyl groups.

The methoxyl group content of bleached CMP acidolysis lignin supports the fact that the isolated lignin is a syringyl-guaiacyl type. The main photodegradation process of phenolic structural elements of lignin is demethoxylation of the guaiacyl or syringyl rings; however, simple phenols might be photooxidized into quinones and muconic acids [11]. Among phenols, guaiacyl elements are more photodegraded than syringyl units. Syringyl elements give very stable phenoxy radicals [23] with a structure related to hindered phenols such as BHT [24]. Guaiacoxy radicals are prone to be transformed in *o*-quinones by demethoxylation. The observed loss of methoxyl groups during photolysis of BCMP lignin has been also noted by examining wood, lignin, and mechanical pulps [25, 26]. Our results are consistent with these findings.

### Determination of the degree of condensation

Condensed structure may arise from radical coupling reactions during irradiation. As the aromatic rings condense in various manners such as 5-5’, β-5, 4-O-5, or other types of linkages, the number of aromatic protons will decrease. Therefore, the condensation degree of the lignin can be calculated from the ^1^H-NMR spectra and the methoxyl contents. For each C_9_ unit, a maximum of four aromatic hydrogen atoms is expected, since the carbons at positions 1 and 4 are linked to the propyl chain and to the phenolic hydroxyl/ether linkage, respectively. The aromatic positions which are occupied by methoxyl groups can be obtained from the C_9_ formula. Therefore, the difference between the four hydrogen atoms and the methoxyl group content gives the theoretical number of aromatic protons. By comparing the actual number of aromatic protons which is obtained from the ^1^H-NMR spectra with this theoretical value, the degree of condensation can be estimated [27, 28]. The degrees of condensation of isolated BCMP acidolysis lignin and the irradiated samples are shown in Table 4. The results indicated that irradiation led to the formation of condensed structures. Increasing phenolic condensed structure with irradiation time has also been reported by Li and Ragauskas [25].

**Table 4:** Condensation degree of unirradiated and irradiated BCMP acidolysis lignin.

	**Irradiation time (hours)**				
	**0**	**2**	**4**	**24**	**120**
**Condensation degree (%)**	10.63	20.23	27.58	41.51	46.83

## Conclusions

Our results indicate that photodegradation of acidolysis lignin isolated from hardwood BCMP is accompanied by a decrease in intensity of a part of the aliphatic proton NMR resonanances, while new signals apperar in the aromatic-olefinic and the aldehyde regions, suggesting that C_α_-C_β_ bond cleavage and formation of benzaldehyde and benzoic acid type compounds could be dominant reactions in the light-induced degradation of BCMP lignin. The formation of stilbenes via opening the hydrofuran ring of phenylcoumaran units may also contribute to the discoloration. In addition, the irradiation caused the number of aromatic ring protons to decrease due to condensation reactions or other mechanisms such as oxidation of aromatic rings to nonaromatic structures. Loss of methoxyl groups was another structural change observed during the photo-degradation of BCMP acidolysis lignin.

## Experimental

### Materials

All reagents and solvents were commercially purchased and used as received. A commercial hardwood CMP bleached with hydrogen peroxide received from Mazandaran Pulp and Paper Ltd. was used in all experiments. The pulp properties were as follows: brightness 71 % ISO; yellowness 26.8 %; yield 83%. Whatman No. 1 filter paper was used as cellulose matrix.

### Isolation of Lignin

Isolation of lignin from BCMP was carried out in accordance with Wang *et al*. [29] and Li *et al*. [25] with slight changes. Prior to isolation of the lignin, the hardwood BCMP was Soxhlet–extracted with acetone for 24 h and the extracted pulp then left to air–dry overnight. Dry pulp (75 g) was then refluxed in a 0.01 N HCl dioxane–water solution (1.5 L, 8.5:1.5) under an argon atmosphere for 1 h. This pulp was filtered and washed with fresh dioxane (100 mL×3). The combined aliquots were neutralized with powdered NaHCO_3_, filtered and concentrated under reduced pressure at 40°C. The concentrated solution was added to 0.006 N aqueous HCl (500 mL) and the precipitated lignin was twice washed with acidified water (pH 2-3) and then freeze–dried. The lyophilized lignin was subsequently dried under high vacuum at room temperature. Mild acidolysis lignins like the ones used in this study are representative of those usually found in mechanical pulp fibers. The lignin preparations were dissolved in a solution of acetic acid-water (90:10 v/v, 1 g of lignin/10 mL of solvent) and then precipitated in ether. After filtration, the precipitated lignin preparations were washed with extra ether and dried under vacuum with P_2_O_5_.

### Filter paper impregnation

The BCMP lignin was dissolved in a 9:1 dioxane-water solution (3 mL) and applied homogeneously to both sides of Whatman filter paper using a syringe. The impregnated test-sheets were dried overnight under partial vacuum over P_2_O_5_. Approximately 21% (based on the weight of the sheet) lignin was impregnated on the filter paper. This amount simulates the lignin content of the hardwood BCMP pulp.

### UV irradiation of lignin treated filter papers

For accelerated exposure, lignin–impregnated sheets were placed in custom–made photoreactor. This reactor was maintained at 30°C and equipped with four 350 nm black lights. Each side of the paper was photolyzed for half of the desired irradiation time.

### Extraction of the photolyzed lignin

The photolyzed filter papers were wrapped to fit into test tubes and were extracted with 9:1 dioxane–water mixture (25 mL × 4). The extract was filtered and then concentrated under reduced pressure at 40°C. The photolyzed lignin samples were freeze–dried and then further dried at room temperature using higher vacuum. This procedure yielded 80-92% of the lignin content of test sheets.

### Acetylation of lignin

According to Lundquist [14], acetylation of lignin samples (100 mg) was performed with acetic anhydride-pyridine (2 mL, 1:1, v/v) at room temperature overnight in a 50 mL flask. Ethanol (25 mL) was added, and after 30 min, the solvents were removed by evaporation under reduced pressure at 40 °C. Addition and removal of ethanol was repeated more than ten times to ensure complete removal of acetic acid and pyridine from the sample.

### Purification of the lignin acetate

The acetate lignin was dissolved in chloroform (2 mL) and the solution was added dropwise to magnetically stirred ether (100 mL). The precipitated acetate was filtered, with ether and then dried under vacuum with P_2_O_5_.

### Quantitative ^1^H-NMR spectroscopy

The quantitative ^1^H-NMR spectra of acetylated lignin samples were recorded on a Bruker Avance 400 MHz spectrometer at room temperature. The samples (60 mg) were accurately weighed and dissolved in CDCl_3_ (0.6 mL). Conditions for analysis included a 90° pulse width, a 1.3 s acquisition time, and a 7 s pulse delay (d1). A relatively high relaxation delay of 7 s was applied to ensure complete relaxation of aldehyde protons, and a total of 128 scans were collected. The spectra were calibrated from the signal of residual chloroform (7.3 ppm).

### Elemental analysis

The carbon and hydrogen determinations of BCMP lignin performed in a CHN-O-Rapide, Elementar analyzer. The oxygen content was calculated by the difference with respect to the total sample. The sulfur content was neglected because it was negligible.

## References

[1] Cockram R.A. (1989). CTMP in Fine Papers. Proc. Intl. Mechanical Pulping Conf., Helsinki, Finland.

[2] Grös G. (1998). Future Prospects for Mechanical Pulp. Pulp Paper Intl..

[3] Agarwal U.P., McSweeny J.D. (1997). Photoyellowing of Thermomechanical Pulps: Looking Beyond α-carbonyl and Ethylenic Groups as Initiating Structures. J. Wood Chem. Technol..

[4] Leary G.J. (1994). Recent Progress in Understanding and Inhibiting the Light-induced Yellowing of Mechanical Pulps. J. Pulp Paper Sci..

[5] Karhunen P., Rummakko P., Sipila J., Brunow G., Kilpeläinen I. (1995). Dibenzodioxocins; A Novel Type of Linkages in Softwood Lignins. Tetrahedron Lett..

[6] Karhunen P., Rummakko P., Sipila J., Brunow G., Kilpeläinen I. (1995). The Formation of Dibenzodioxocin Structure by Oxidative Coupling. A Model Reaction for Lignin Biosynthesis. Tetrahedron Lett..

[7] Karhunen P., Rummakko P., Pajunen A., Brunow G. (1996). Synthesis and Crystal Structure Determination of Model Compounds for the Dibenzodioxocine Structure Occurring in Wood Lignins. J. Chem. Soc., Perkin Trans. 1.

[8] Gardrat C., Ruggiero R., Hoareau W., Nourmamode A., Grelier S., Siegmud B., Castellan A. (2004). Photochemical Study of an O-ethyl Dibenzodioxocin Molecule as a Model for the Photodegradation of Non-phenolic Lignin Units of Lignocellulosics. J. Photochem. Photobiol. A: Chem..

[9] Gardrat C., Ruggiero R., Hoareau W., Damigo L., Nourmamode A., Grelier S., Castellan A. (2005). Photochemical Study of 4-(4,9-dimethoxy-2,11-n-dipropyl-6,7-dihydro-5,8-dioxadibenzo[a,c]-cycloocten-6-yl)-2-methoxyphenol, a Lignin Model of Phenolic Dibenzodioxocin Unit. J. Photochem. Photobiol. A: Chem..

[10] Argyropoulos D.S., Argyropoulos D.S. (1995). Applications of Quantitative ^31^P NMR to Pulping, Bleaching and Yellowing. Advances in Lignocellulosics Characterization.

[11] Castellan A., Nourmamode A., Jaeger C., Forsskăhl I., Heitner C., Scaiano J.C. (1993). Photochemistry of Quinones and Hydroquinones in Solid 2-hydroxypropylcellulose Films and on Filter Paper. Photochemistry of Lignocellulosic Materials.

[12] Agarwal U.P. (1998). Assignment of the Photoyellowing-related 1657 cm^-1^ Raman/IR Band to p-quinones and Its Implications to the Mechanism of Color Reversion in Mechanical Pulps. J. Wood Chem.Technol..

[13] Azadfallah M., Mirshokraei S.A., Latibari A.J., Parsapajouh D. (2008). Analysis of Photodegraded Lignin on Cellulose Matrix by Means of FTIR Spectroscopy and High Pressure Size Exclusion Chromatography. Iran Polym. J..

[14] Lundquist K., Lin S.Y. Dence C.W. (1992). Methods in Lignin Chemistry.

[15] Ludwig C.H., Nist B.J., McCarthy J.L. (1964). Lignin. XIII. The High Resolution Nuclear Magnetic Resonance Spectroscopy of Protons in Acetylated Lignins. Am. Chem. Soc..

[16] Chen C.-L., Robert D., Wood W.A., Kollog S.T. (1988). Methods in Enzymology.

[17] Holmbom B., Ekman R., Eckerman C. (1989). Degradation Products Formed during Light- and Heat-induced Yellowing of Spruce Groundwood. Proc. Intl. Wood Pulp. Chem. Atlanta, USA.

[18] Chan F.D., Nguyen K., Wallis A.F.A. (1995). Contribution of Lignin Sub-structures to Nitrobenzene Oxidation Products. J. Wood Chem. Technol..

[19] Sjöholm R., Holmbom B., Akerback N. (1992). Studies of the Photodegradation of Spruce Lignin by NMR Spectroscopy. J. Wood Chem. Technol..

[20] Lin S.Y., Dence C.W., Lin S.Y., Dence C.W. (1992). Methods in Lignin Chemistry.

[21] Baez J., Freer J., David N.S.H., Shiraishi N. (2001). Wood and Cellulose Chemistry.

[22] Argyropoulos D.S., Sun Y. (1996). Photochemically Induced Solid State Degradation, Condensation, and Rearrangement Reactions in Lignin Model Compounds and Milled Wood Lignin. Photochem. Photobiol..

[23] Steelink C. (1965). Stable Phenoxy Radicals Derived from Phenols Related to Lignin. J. Am. Chem. Soc..

[24] Barclay L.R.C., Xi F., Norris J.Q. (1997). Antioxidant Properties of Phenolic Lignin Model Compounds. J. Wood Chem. Technol..

[25] Li C., Ragauskas A. J. (1999). Brightness Reversion of Mechanical Pulps. Part XIII: Photoinduced Degradation of Lignin on Cellulose Matrix. J. Wood Chem. Technol..

[26] Leary G.J. (1967). The Yellowing of Wood by Light. Tappi J..

[27] Adilson R., Goncalves U.S., Maria L.B., Antonio A.S.C. (2000). Piassava Fibers (Attalea funifera): NMR Spectroscopy of Lignin. J. Braz. Chem. Soc..

[28] Bland D. E., Strenhell S. (1965). Estimation of Aromatic Protons in Methanol Lignins of Pinus Radiata and Eucalyptus Regnans from Proton Magnetic Resonance Spectra. Aust. J. Chem..

[29] Wang J., Jiang Z.H., Argyropoulos D.S. (1997). Isolation and Characterization of Lignin Extracted from Softwood Kraft Pulp after Xylanase Treatment. J. Pulp Paper Sci..

